# Nonpharmacological Interventions for Pain Relief During Peripheral Venous Cannulation: Implications for Practice

**DOI:** 10.3390/jcm15072662

**Published:** 2026-03-31

**Authors:** Damian Romańczuk, Aleksandra Maruszak, Sandra Lange, Wioletta Mędrzycka-Dąbrowska, Grzegorz Cichowlas, Anna Gąsior

**Affiliations:** 1 Department of Anesthesiology and Intensive Care, Medical University of Gdańsk, 80-952 Gdańsk, Poland; damian.romanczuk@gumed.edu.pl; 2 Department of Anesthesiology and Intensive Care, University Clinical Center, 80-952 Gdańsk, Poland; a.maruszak@gumed.edu.pl; 3 Department of Internal and Pediatric Nursing, Medical University of Gdańsk, 80-211 Gdańsk, Poland; langa94@gumed.edu.pl; 4 Department of Anesthesiology and Intensive Care Education, Medical University of Warsaw, 02-097 Warsaw, Poland; grzegorz.cichowlas@wum.edu.pl; 5 Department of Anesthesiology and Intensive Care, Czerniakowski Hospital sp. z.o.o., 00-739 Warsaw, Poland; anna.gasior@szpitalczerniakowski.waw.pl

**Keywords:** peripheral venous cannulation, pain management, non-pharmacological interventions, distraction, procedural pain, nursing care

## Abstract

**Background:** Peripheral venous cannulation is one of the most common clinical procedures, yet it often causes significant pain, anxiety, and discomfort for patients. While pharmacological methods exist, non-pharmacological interventions offer a low-cost, low-risk alternative that eliminates waiting times for anesthetic onset. The aim of this review is to synthesize the various nonpharmacological interventions for procedural pain reduction during PIVC in adults, covering interventions ranging from psychological distraction to advanced procedural support technologies. **Methods:** A systematic review was conducted following PRISMA 2020 guidelines and the Joanna Briggs Institute (JBI) framework. Databases including PubMed, CINAHL, Web of Science, and Scopus were searched for studies published between 2015 and 2025. Inclusion criteria focused on randomized controlled trials (RCTs) and quasi-experimental studies involving adult patients undergoing PIVC. **Results:** Thirty studies (29 randomized controlled trials and one experimental study) were included in the final analysis. The interventions were categorized into three primary groups: distraction techniques, physical methods, and behavioral techniques. The application of virtual reality (VR), optical illusion cards, and music therapy significantly reduced pain scores and enhanced patient satisfaction. Similarly, physical methods, such as thermomechanical stimulation (e.g., the Buzzy^®^ device), local heat application, and vibration, were found to be effective in lowering pain intensity compared to standard care. Behavioral techniques, including the “cough trick,” diaphragmatic breathing, and the Valsalva maneuver, consistently demonstrated efficacy in reducing both procedural pain and anxiety. Notably, while most interventions successfully reduced pain, certain methods—such as near-infrared (NIR) vein visualization—improved procedural success rates without significantly altering the subjective perception of pain. **Conclusions:** Findings from this review suggest that non-pharmacological interventions may serve as effective, safe, and feasible adjuncts for pain management during peripheral venous cannulation. Techniques such as the cough trick and vibration-based devices are particularly recommended due to their ease of integration into routine nursing practice, potentially improving patient comfort and clinical outcomes.

## 1. Introduction

Securing a peripheral intravenous catheter (PIVC) is essential for safe anesthesia administration, and 70–80% of hospitalized patients undergo at least one insertion. Adults report that this procedure is painful and stressful [1,2]. Patients who may require peripheral intravenous access include those with dehydration, severe illness, need for rapid fluid resuscitation, electrolyte disturbances, or long-term drug therapy [3]. Fear of this procedure can trigger an autonomic response resulting in vasoconstriction [4]. In many cases, successful catheterization requires multiple insertions. These subsequent attempts can cause pain and delay the initiation of diagnostic procedures or investigations. Furthermore, multiple insertions can lead to vessel wall degradation, complicating subsequent insertions and thus increasing the risk of infection [5]. Appropriate analgesic management plays a crucial role in improving patient comfort, improving the overall hospital experience, and facilitating better patient–provider cooperation [1,2]. Insertion of the catheter is the responsibility of nurses and should be performed safely [3]. Based on studies assessing pain associated with intravenous cannulation, antecubital fossa (ACF) insertion is usually preferred over dorsum of the hand (DOH) to minimize patient pain. The density of sensory innervation in the skin varies depending on the site; therefore, pain experienced at different sites is likely to vary [6,7]. Some studies suggest that the size of the catheter itself, ranging from 14G to 22G, is not a key factor in pain. Other parameters, such as gender, site of insertion, and success rate of the first attempt, are more important [6]. For PIVC application, it is recommended to prefer areas that do not restrict the patient’s movement and enable participation in self-care when necessary, minimizing the risk of catheter dislodgement or obstruction and pain [8].

In clinical practice, both pharmacological and nonpharmacological methods are used to divert attention and minimize the pain associated with cannulation [1,2]. Non-pharmacological methods of bringing pain under control are one of the complementary elements in the approach to the comprehensive reduction of pain. Non-pharmacological methods used in treatment have a greater effect on the emotional, cognitive, behavioral and sociocultural aspects of pain. Also, non-pharmacological methods are low-risk, cost less, and are practicable and easy to apply, meaning they constitute a complementary element in the approach to pain reduction [2].

### Aim

The aim of this review is to synthesize the various nonpharmacological interventions for procedural pain reduction during PIVC in adults, covering interventions ranging from psychological distraction to advanced procedural support technologies. The research questions were as follows:

Q1: Which categories of non-pharmacological interventions methods (specifically distraction, physical, and behavioral techniques) are most effective in the adult population?

Q2: What is the clinical effectiveness of various non-pharmacological interventions in reducing procedural pain during PIVC?

Q3: How do these interventions affect secondary outcomes such as patient anxiety and satisfaction?

Q4: What is the impact of non-pharmacological interventions on psychosocial and procedural outcomes in adult patients undergoing peripheral venous cannulation?

## 2. Methods

### 2.1. Study Design

The methodological framework for this systematic review was established based on the Joanna Briggs Institute (JBI) critical appraisal tools [9,10]. Furthermore, the study was designed and reported in strict accordance with the Preferred Reporting Items for Systematic Reviews and Meta-Analyses (PRISMA) 2020 guidelines [11] (Supplementary Materials) to ensure the transparency and high quality of the evidence synthesis.

### 2.2. Search Strategy

The following electronic databases were searched: PubMed, CINAHL, Web of Science, and Scopus. The search strategy employed a combination of keywords and MeSH terms, including: “adults”, “outpatients”, “inpatients”, “peripheral catheterization”, “venipuncture”, “peripheral intravenous catheter”, “PIVC”, “IV insertion”, “pain management”, “analgesia”, and “non-pharmacological interventions”. These terms were combined using Boolean operators AND and OR. Detailed search strings for each database are documented in Table 1.

The inclusion period was limited to studies published between 2015 and 2025, with the final search updated in January 2026. The initial screening of titles and abstracts was performed independently by three reviewers (DR, GC, and AG) to identify duplicates and irrelevant records. Subsequently, the full-text versions of potentially eligible articles were retrieved and critically appraised based on the PICO framework and predefined inclusion/exclusion criteria. The PICO framework and the eligibility criteria are summarized in Table 2 and Table 3, respectively.

Initial screening of titles and abstracts for eligibility was performed independently by two authors. Subsequently, a critical appraisal of the full-text articles was conducted to verify their compliance with the established inclusion criteria. In cases of disagreement regarding study qualification, a final decision was reached through mutual discussion or by seeking the opinion of a third expert.

### 2.3. Data Extraction

Data were independently extracted by two reviewers (DR and WMD) using a standardized data extraction form. The following information was collected from each study: first author and year of publication, study design, sample size, clinical setting, type and detailed description of the intervention, primary and secondary outcome measures (including pain assessment tools), and key clinical findings. Any discrepancies between the reviewers were resolved through consensus or by consulting a third member of the research team. The data extraction template was not pilot tested, but all reviewers used a shared coding manual to ensure reliability.

### 2.4. Data Synthesis

Due to the substantial heterogeneity among the included studies—specifically regarding the diversity of non-pharmacological interventions, varied clinical settings, and the use of different pain assessment tools (e.g., VAS, NRS, Faces Pain Scale)—a quantitative meta-analysis was deemed inappropriate. Pooling such heterogeneous data would introduce a significant risk of bias and lead to unreliable clinical conclusions. Consequently, a structured narrative synthesis was performed [9,10]. The findings were categorized thematically to provide a comprehensive mapping of the evidence, with key data synthesized in a summary table to highlight the clinical efficacy, safety, and feasibility of each intervention in adult populations.

### 2.5. Quality Assessment

The methodological quality of the included studies was independently evaluated by two tools: the Checklist for Randomized Controlled Trials and the Checklist for Quasi-Experimental Studies [9,10]. Study quality was classified into three categories: high, moderate, and low (Table 4 and Table 5). A point was awarded only when the response to a specific question in the checklist was “Yes.” Any disagreements between reviewers were resolved through discussion until a consensus was reached.

Q1—Was true randomization applied for assigning participants to treatment groups? Q2—Was the allocation to treatment groups adequately concealed? Q3—Were the treatment groups comparable at baseline in terms of key characteristics? Q4—Were participants blinded to their assigned treatment group? Q5—Were the individuals administering the intervention blinded to group allocation? Q6—Were outcome assessors blinded to the participants’ group assignment? Q7—Apart from the intervention under study, were the treatment groups managed identically? Q8—Was follow-up complete, and if not, were differences in follow-up between groups appropriately described and analyzed? Q9—Were participants analyzed in the groups to which they were originally randomized? Q10—Were outcomes measured consistently across all treatment groups? Q11—Were outcome measurements performed in a valid and reliable manner? Q12—Was an appropriate statistical approach used for data analysis? Q13—Was the study design suitable, and were any deviations from the standard RCT framework (e.g., individual randomization, parallel groups) properly addressed in the conduct and analysis of the trial?

Q1—Does the study clearly distinguish the “cause” from the “effect,” ensuring there is no ambiguity about which variable occurs first? Q2—Were the participants included in the comparisons comparable in key characteristics? Q3—Did participants in the comparison groups receive similar care or treatment, aside from the intervention or exposure under investigation? Q4—Was there a control group included in the study? Q5—Were outcome measures collected at multiple time points, both before and after the intervention or exposure? Q6—Was follow-up complete, and if not, were any differences in follow-up between groups adequately described and analyzed? Q7—Were the outcomes of participants in the comparison groups measured in a consistent manner? Q8—Were the outcome measurements conducted in a reliable and valid way? Q9—Was an appropriate statistical analysis applied to the data?

### 2.6. Ethical Approval

As this study was conducted as a systematic literature review and involved the analysis of previously published data, formal ethical approval and patient informed consent were not required.

## 3. Results

### 3.1. Study Selection

The initial database search yielded a total of 361 articles. Following the removal of 22 duplicates, 312 records were excluded during the title and abstract screening phase. A total of 35 full-text articles were assessed for eligibility; of these, 5 studies were excluded due to inappropriate population, setting, context, or study design.

**Figure 1:**
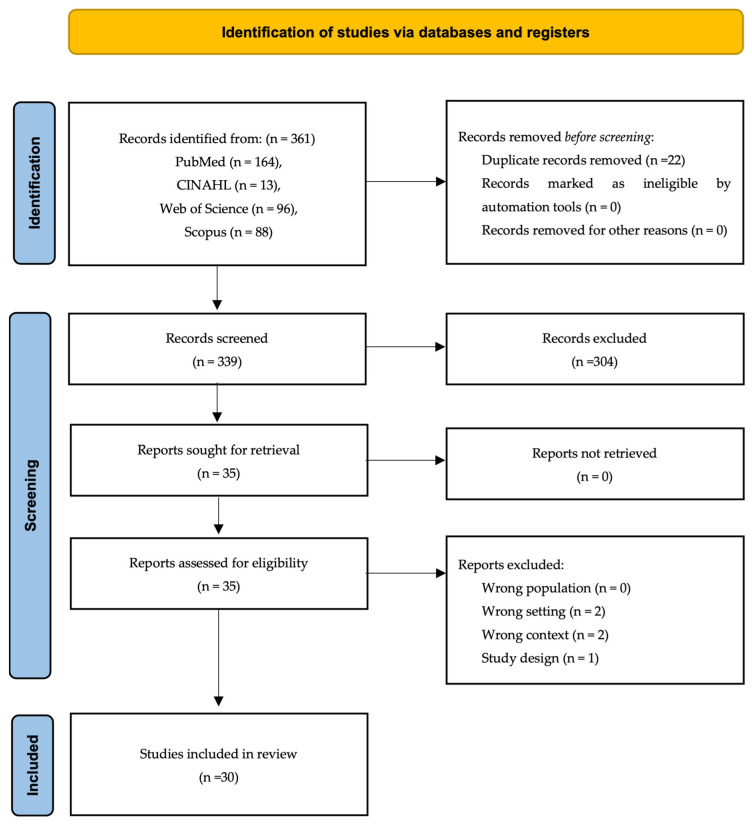
PRISMA 2020 flow diagram of study selection [10].

Ultimately, 30 studies met the inclusion criteria and were included in the final analysis (Figure 1). The synthesized evidence comprises 29 randomized controlled trials and one experimental study.

### 3.2. Characteristics of Included Studies

Based on the analysis of the thirty publications included in this systematic review, a detailed characterization of the study populations and clinical settings was conducted. The sample sizes in the analyzed reports showed considerable variation, reflecting the methodological diversity and the specific nature of the interventions studied. The total number of participants (N) in individual studies ranged from 59 to 272, with the majority of studies maintaining a sample size between 100 and 150 patients. The size of individual groups—both intervention (IG) and control (CG)—typically ranged from 30 to 65 participants, ensuring adequate statistical power for the analyses. In multi-arm studies comparing the effectiveness of several different non-pharmacological techniques (e.g., simultaneous analysis of the “cough trick,” incentive spirometry, and stress balls), the size of individual study arms remained relatively constant, averaging between 29 and 33 subjects.

Geographically, the studies were distributed across 12 countries, with the highest concentration in Turkey (n = 15). Other contributions included France (n = 3), Spain (n = 2), Iran (n = 2), South Korea (n = 2), and India (n = 2). The remaining studies were conducted in Germany (n = 1), the USA (n = 1), Switzerland (n = 1), Italy (n = 1), Brazil (n = 1), and Belgium (n = 1). Notably, one of the European records was a multicenter study conducted jointly in France and Belgium.

These studies were conducted across a variety of healthcare units, enabling an assessment of intervention efficacy in diverse clinical contexts. Key observation sites included Emergency Departments (ED) and urgent care units (yellow and green zones), where high-paced workflows necessitate rapid and effective pain management methods. Furthermore, procedures were performed in general surgery clinics, outpatient oncological chemotherapy wards, as well as specialized treatment rooms and blood donation centers. A summary of the key findings from the included studies is provided in Table 6.

### 3.3. Non-Pharmacological Interventions Recommended for Adult Patients Undergoing Peripheral Vascular Cannulation

**Figure 2:**
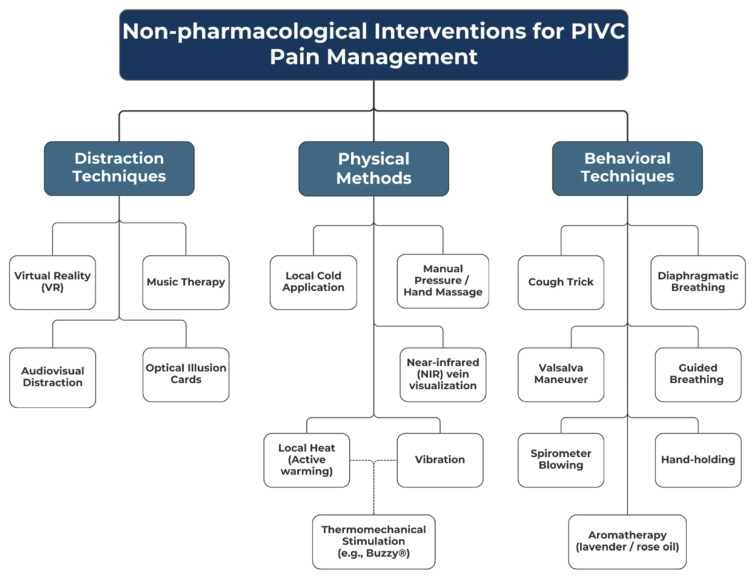
Non-pharmacological interventions for PIVC pain management.

Based on a systematic review of 30 clinical studies (comprising 29 randomized controlled trials and one experimental study), several effective non-pharmacological interventions were identified. Following the established methodology, these interventions were organized into a conceptual framework consisting of three primary domains: distraction techniques, physical methods, and behavioral interventions (Figure 2).

Distraction Techniques These methods aim to redirect the patient’s attention away from the painful stimulus by engaging other senses and cognitive processes. The following interventions were evaluated:

- Virtual Reality (VR): The most advanced form of immersive distraction, effectively isolating the patient from the clinical environment [14,20,22]
- Optical Illusion Cards: The use of visual stimuli that require cognitive focus and engagement [14].
- 3D Videos and Audiovisual Stimulation: Engaging visual and auditory pathways to reduce procedural anxiety and pain perception [14,22].

Physical Methods These interventions involve direct physical action at the puncture site or the utilization of physiological mechanisms to inhibit pain transmission:

- Local Thermal Therapy (Active Warming): Warming the puncture site to induce vasodilation and reduce pain [21,29,31,37].
- Cryotherapy (Cold Application): Use of ice packs or cooling sprays to produce local anesthesia [18,29].
- Vibration: Application of vibrating devices to interfere with the transmission of pain impulses [18].
- Thermomechanical Stimulation (Buzzy^®^ device): Simultaneous application of vibration and cooling, based on the Gate Control Theory of Pain [13]. As illustrated in Figure 2, this method integrates both mechanical and thermal components (vibration and cold) to achieve synergistic analgesia.

Behavioral Techniques and Physical Maneuvers Simple interventions based on patient cooperation and the modification of physiological responses:

- The “Cough Trick”: Performing a short, controlled cough at the exact moment of needle insertion [17,42].
- Breathing Techniques: Diaphragmatic breathing or rhythmic breath-holding to reduce muscle tension and procedural stress [15,20].
- Valsalva Maneuver: Forced expiration against a closed glottis [27,32,39].
- Stress Ball Squeezing: Activation of forearm muscles combined with mechanical distraction [17,19,20].
- Acupressure: Application of localized pressure at the puncture site prior to the procedure [21].

The implementation of these methods represents a low-cost, safe, and effective alternative to pharmacological agents, allowing for immediate procedural execution without the delay associated with the onset of local anesthetics.

### 3.4. Pain Intensity During Peripheral Vascular Cannulation

A systematic analysis of the identified non-pharmacological strategies revealed a significant reduction in pain intensity across the majority of evaluated interventions. Procedural pain was primarily assessed using standardized subjective scales: Visual Analog Scale (VAS)—utilized in 20 studies. Numerical Rating Scale (NRS)—employed in 10 studies.

Physical methods, largely based on the Gate Control Theory of Pain, demonstrated high efficacy in mitigating procedural discomfort. The following raw data represent key findings:

- Thermomechanical Stimulation (Buzzy^®^ device): In a study by Cetin [13], the mean pain score in the Buzzy group was 1.04 ± 0.96 cm (VAS), compared to 5.32 ± 1.64 cm (VAS) in the control group. Similarly, Serin [22] reported scores of 2.66 ± 1.22 (VAS) for the intervention group versus 4.85 ± 1.71 (VAS) for the control.
- Cryotherapy: Pre-procedural application of cold packs reduced pain to 30.50 ± 16.78 mm (VAS), compared to 48.36 ± 24.02 mm (VAS) in the standard care group [18].
- Heat and Pressure: The application of high pressure (100 mmHg) combined with heat achieved a clinically significant pain reduction of at least 1 point on the VAS [21].
- Vibration: While effective when combined with cold (Buzzy^®^), isolated vibration did not always yield statistically significant results (e.g., 56.66 ± 14.03 mm (VAS) in the vibration group vs. 48.36 ± 24.02 mm (VAS) in the control) [18].

Technological distraction methods significantly reduced the patient’s cognitive focus on the painful stimulus:

- Virtual Reality (VR): Rişvan [21] observed pain levels of 1.94 ± 0.40 (NRS) in the VR group versus 2.80 ± 0.36 (NRS) in the control group. Serin [24] reported 2.71 ± 1.45 (VAS) for VR compared to 4.85 ± 1.71 (VAS) in the control.
- Optical Illusion Cards and 3D Video: Distraction groups reported mean scores of 3.32–3.50 ± 2.81–2.84 (VAS), whereas the control group reported 4.72 ± 3.15 (VAS) [14].

Simple behavioral and physical maneuvers also provided measurable analgesic benefits:

- Cough Trick: This method resulted in pain scores of 19.5 mm (VAS) compared to 45.5 mm (VAS) in the control group [17].
- Breathing Exercises: Diaphragmatic breathing significantly reduced pain to 0.29 ± 0.70 (NRS), whereas the control group score was 1.30 ± 1.47 (NRS) [15].
- Stress Ball Squeezing: This intervention yielded scores of 0.85 ± 0.23 (NRS) versus 2.80 ± 0.36 (NRS) in the control group [19].

### 3.5. Statistical Significance and Effect Size of Non-Pharmacological Interventions

The systematic review of the included studies demonstrated that non-pharmacological interventions consistently yielded statistically significant reductions in pain intensity (*p* < 0.05). The clinical significance of these findings was further supported by the calculation of effect sizes, primarily using Cohen’s, which allowed for a standardized comparison of the interventions’ impact.

Quantitative Pain Reduction Analysis

The most pronounced differences were observed in interventions utilizing high-tech distraction and thermomechanical stimulation:

- Thermomechanical Stimulation (Buzzy^®^): In studies such as those by Cetin [15] and Serin [24] the intervention groups reported mean pain scores ranging from 1.04 to 2.66 (NRS/VAS), while control groups consistently reported scores between 4.85 and 5.32. This represents a reduction in pain intensity of approximately 50–70%.
- Virtual Reality (VR): Studies by Rişvan [19] and Serin [22] showed that VR reduced pain levels to VAS < 2.0, compared to VAS 3.5–5.0 in control groups.

Studies Reporting Cohen’s (Effect Size)

Several studies specifically calculated Cohen’s to quantify the magnitude of the intervention’s effect:

- Large Effect Size (d > 0.8): * Virtual Reality (VR): In the studies by Rişvan (2025) [19] and Serin (2025) [22] immersive VR interventions demonstrated large effect sizes (often exceeding), indicating a robust clinical benefit.
- Thermomechanical Stimulation (Buzzy^®^): Research by Cetin (2019) [13] and Serin (2025) [22] reported large effect sizes for the combination of vibration and cold, confirming it as one of the most effective physical methods.
- Medium Effect Size (d = 0.5–0.8): Active Warming: Studies evaluating localized heat showed a medium effect size, proving effective for both pain reduction and improved vein visibility.
- Music Therapy and Valsalva Maneuver: These interventions Tapar [39] generally yielded medium effect sizes.
- Small Effect Size (d = 0.2–0.5): * Isolated Vibration and Passive Distraction: In the study by Esra Yılmaz [18] passive methods or isolated mechanical vibration showed smaller effect sizes, suggesting they may be less effective when used as standalone treatments for patients with high procedural anxiety.

### 3.6. Impact of Non-Pharmacological Modalities on Psychosocial and Procedural Outcomes

The systematic review identified several secondary benefits of non-pharmacological interventions that extend beyond analgesia, particularly concerning patient anxiety, procedural efficiency, and clinical safety. Immersive technologies, specifically Virtual Reality (VR), demonstrated a unique capacity to attenuate procedural anxiety; by inducing a state of presence within a virtual environment, VR effectively decoupled psychological distress from physical stimuli, fostering superior emotional stability compared to standard care [26]. Furthermore, thermal interventions provided distinct clinical advantages in terms of procedural efficiency. Active warming served not only as a localized analgesic but also significantly enhanced peripheral venous visibility and palpability through induced vasodilation. This physiological response directly correlated with increased first-attempt cannulation success rates, thereby optimizing resource consumption and reducing overall procedural time [22]. From a patient-centered perspective, mechanical interventions—such as the Buzzy^®^ device—were associated with high levels of perceived control and superior satisfaction scores. Due to their non-invasive nature and immediate onset of action, patients expressed a strong preference for these modalities in future clinical encounters, specifically to mitigate the “needle-phobia” often exacerbated by injectable anesthetics [19,23]. Finally, the safety profile of these interventions remained exemplary across all 30 reviewed studies. The absence of adverse events, such as skin irritation, prolonged vasoconstriction, or neurological discomfort, confirms the high feasibility of integrating non-pharmacological methods into both acute and outpatient nursing workflows.

## 4. Discussion

The primary objective of this systematic review was to identify and evaluate the clinical efficacy of non-pharmacological interventions aimed at reducing pain during peripheral venous cannulation (PVC) using SPC in adult patients. The synthesis of 30 clinical trials confirms that PVC remains a clinically relevant source of procedural pain and distress in adults. However, the inclusion of interventions with diverse clinical purposes—ranging from psychological distraction to procedural support technologies (e.g., vein visualization)—introduces a degree of conceptual heterogeneity. While all included methods are applied during PIVC, their primary targets differ; some aim to modulate pain perception directly, while others focus on procedural efficiency, which may only indirectly affect the patient’s sensory experience. This diversity necessitates a cautious interpretation, as the overall ‘effectiveness’ of non-pharmacological methods cannot be viewed as a monolithic effect, but rather as a spectrum of outcomes depending on the specific mechanism of the intervention. The findings suggest that several non-pharmacological approaches, including mechanical stimulation and distraction-based techniques, may contribute to pain reduction during the procedure. Overall, the available evidence highlights the potential role of these interventions in improving patient comfort during routine cannulation.

A key finding of this review is the necessity of a multifaceted approach to procedural pain. We categorized the interventions into those directly modulating pain perception (e.g., cryotherapy, VR) and those providing procedural guidance (e.g., USG, NIR). It is crucial to highlight that although ultrasound and near-infrared visualization are primarily technical aids, they function as vital indirect non-pharmacological analgesic tools. By increasing the first-attempt success rate and reducing the total number of needle passes, these technologies minimize mechanical tissue trauma and cumulative nociceptive input. This confirms that for vascular visualization techniques, the effects on pain reduction appear through indirect pathways—primarily by increasing procedural efficiency and reducing the number of failed attempts—rather than through direct modulation of pain receptors. Therefore, improving procedural precision should be considered an integral part of a holistic pain-reduction strategy in clinical nursing.

Peripheral intravenous cannulation is a painful and stressful experience for patients, requiring routine use of pain relief methods [42,43]. The authors propose a person-centered care model in which the patient actively participates in decisions such as the choice of insertion site. Improving the patient experience requires not only analgesia, but also dialog and technical strategies (e.g., the use of ultrasound for difficult access) that increase the effectiveness of the first attempt and minimize procedural pain [43,44,45]. Our review shows that the routine reduction of pain and greater involvement of patients in decisions, as requested by patients, can be realistically implemented through simple, low-cost non-pharmacological techniques and strategies to increase the effectiveness of the first attempt, thereby reducing the number of repeat punctures and cumulative procedural pain.

Regarding the efficacy of Mechanical and Technological Interventions, a prominent finding in this review is the high analgesic potency of thermomechanical stimulation and immersive technologies. The Buzzy^®^ device, which integrates high-frequency vibration with cryotherapy, demonstrated superior pain reduction across multiple settings, with some studies reporting a decrease in VAS scores from 5.32 in control groups to as low as 1.04 in intervention groups. This physiological effect is primarily rooted in the Gate Control Theory, where the vibration and cold sensations travel along faster nerve fibers, effectively “closing the gate” to pain signals, a mechanism similarly effective in mitigating the sharp pain of chest tube removal [46]. Furthermore, the application of local cryotherapy has been successfully utilized to reduce the intense pain associated with arterial blood gas (ABG) sampling, a procedure notoriously more distressing than standard venous access [47]. While most data support its use, the variability in outcomes noted by some researchers suggests that the device’s efficacy may be most pronounced in patients presenting with high pre-procedural anxiety. Similarly, VR has emerged as a robust cognitive intervention. By fostering a state of immersion, VR goggles effectively compete for the brain’s limited attentional resources. This immersive distraction has proven equally effective in managing high-intensity pain during burn wound care and bone marrow biopsies [48,49] offering a more immediate and versatile alternative to the localized, time-dependent effects of pharmacological agents [50].

One of the most clinically relevant insights from this analysis is the efficacy of behavioral maneuvers. Techniques such as the “cough trick,” [14,17,20] diaphragmatic breathing [15], and the use of stress balls consistently [17,19,20] achieved statistically significant pain reduction. For instance, the cough-trick was found to be more effective than simple distraction, likely due to the activation of segmental pain inhibitory pathways and the endogenous opioid system, as suggested by recent experimental evidence where the analgesic effect of coughing was diminished under opioid antagonist blockade [42]. Unlike pharmacological analgesia, which primarily targets pain perception, active warming [21,37] as well as procedural guidance technologies (USG, NIR) may offer an additional procedural benefit by improving vein visibility and palpability. It can be considered a useful option when technical cannulation conditions are suboptimal. The use of warm compresses before cannulation can have a dual clinical benefit. Local heat application promotes vasodilation, which increases the visibility and palpability of vessels and, consequently, increases the likelihood of successful cannulation on the first attempt [44]. This is important because the number of cannulation attempts is an independent factor influencing the pain experience. Multiple attempts are associated with higher pain ratings. In practice, this means that efforts to reduce procedural pain should include not only analgesic interventions (pharmacological and non-pharmacological) but also strategies to improve the technical conditions of cannulation, which reduce the risk of repeated cannulations and lessen the patient’s exposure to pain [46,48,49,50]. The methods presented are low-cost, do not generate additional financial outlays, or do not require specialized equipment or lengthy training of staff or patients. Their implementation in clinical practice is relatively simple and can be carried out routinely as part of standard preparation for cannulation.

### Impact on Psychosocial Outcomes and Safety

In alignment with the secondary research questions, non-pharmacological interventions demonstrated a profound impact on anxiety and patient satisfaction. Modalities such as aromatherapy (lavender and rose oil) [30,36] and hand massage [25], while occasionally showing modest effects on acute physical pain, were highly effective in stabilizing vital signs and lowering procedural stress. These effects are primarily attributed to the stimulation of the limbic system via olfactory pathways and the activation of pressure receptors that trigger a parasympathetic response, effectively reducing the release of stress hormones like cortisol [51]. This psychological stabilization is particularly vital for patients requiring frequent invasive access, such as those undergoing hemodialysis, where music intervention has been shown to significantly decrease both pain intensity and procedural distress during repeated fistula needle insertions [52]. In this regard, as highlighted by Wujtewicz M. et al. [53], the integration of objective physiological markers, such as heart rate variability (HRV), could serve as a crucial tool for the robust validation of the stress-reducing effects of non-pharmacological interventions in clinical practice [53]. In future research, the inclusion of objective physiological stress markers, such as heart rate variability or cortisol levels, would further strengthen the clinical robustness and validity of the findings. Across all included studies, these interventions maintained an exemplary safety profile with no reported adverse events, reinforcing their feasibility for routine integration into nursing practice.

While some interventions, such as aromatherapy or specific distraction techniques, were supported by a limited number of studies, their inclusion in this review serves to map the full landscape of current non-pharmacological possibilities. However, it is crucial to emphasize that the limited body of evidence in these specific categories significantly impacts the generalizability of the reported effects. These findings should be interpreted as preliminary, as the small sample sizes and the paucity of multi-center trials in certain intervention groups restrict our ability to draw definitive clinical conclusions. Consequently, while these methods show promise, their routine implementation should be approached with caution until supported by further high-quality randomized controlled trials.

These results suggest that while some methods are highly promising, others require further validation. To facilitate the interpretation of these heterogeneous findings, we have summarized the strength and consistency of the evidence for each intervention category in Table 7 below.

## 5. Limitations

Several methodological constraints must be acknowledged when interpreting the findings of this systematic review.

- A significant limitation of this review is the varying level of evidence strength across the different intervention categories. While some modalities, such as thermomechanical stimulation (e.g., the Buzzy^®^ device) [13,15,18] and virtual reality (VR) [21,24], are supported by multiple robust clinical trials, the evidence for other interventions remains preliminary. For instance, techniques such as aromatherapy [28,34], specific types of illusion cards [14], or certain behavioral maneuvers [34] are often based on a limited number of studies with small sample sizes. Consequently, findings regarding these specific modalities should be interpreted with caution, as their clinical effectiveness cannot be definitively established at this stage. Further high-quality randomized controlled trials (RCTs) are urgently needed to validate these initial findings and provide more robust data to support their routine implementation in clinical practice.
- Methodological Heterogeneity: The primary limitation arises from the significant diversity in study designs, intervention protocols (e.g., varying durations of vibration or heat application), and the diverse clinical settings (emergency vs. elective departments). Such heterogeneity complicates the direct comparison of effect sizes across all 30 studies.
- Risk of Performance Bias: Due to the physical nature of non-pharmacological interventions (e.g., VR goggles, Buzzy^®^ devices, or breathing exercises), double blinding of participants and healthcare providers was virtually impossible in the majority of the included trials. This inherent lack of blinding may increase the risk of performance and detection bias. To mitigate this, future research should consider the use of ‘sham’ control conditions (e.g., inactive vibration devices) and incorporate objective physiological markers of stress, such as heart rate variability, to complement subjective pain reports.
- Subjectivity of Assessment: The reliance on subjective scales, such as VAS and NRS, introduces potential variability in pain reporting, which is influenced by individual pain thresholds and psychological factors.
- Generalizability and Systems Constraints: A significant proportion of the analyzed studies were conducted in diverse international healthcare settings, which may limit the direct generalizability of the findings across different clinical environments. The transferability of these results is potentially influenced by systemic variations in nursing competencies, the availability of specialized equipment, and differences in institutional organizational protocols. Consequently, the universal application of these non-pharmacological modalities requires careful consideration of local healthcare frameworks and resource availability.
- Statistical Limitations: The absence of a registered protocol in PROSPERO and the lack of a quantitative meta-analysis (including assessments of heterogeneity or publication bias through funnel plots) limit the ability to provide a singular, pooled estimate of effect for the analyzed interventions.

## 6. Conclusions

Peripheral venous cannulation in the adult population is frequently associated with moderate pain intensity, with mean scores often ranging from 3–6 on NRS. These data suggest that PIVC should not be classified as a pain-neutral procedure; therefore, the integration of routine analgesic or distractive interventions should be considered to maintain clinical standards of patient comfort. Based on the reviewed studies, non-pharmacological modalities, specifically thermomechanical stimulation (e.g., the Buzzy^®^ device) and Virtual Reality (VR), demonstrate potential clinical efficacy, in some cases exceeding the Minimal Clinically Important Difference (MCID) and providing a localized analgesic effect that may be comparable to conventional pharmacological agents.

Notably, local thermal therapy (active warming) may provide a synergistic advantage by reducing pain while simultaneously inducing peripheral vasodilation, thereby potentially increasing the success rate of first-attempt cannulation. In this context, procedural guidance techniques, such as ultrasound (USG) or near-infrared visualization, also may play a significant role in the non-pharmacological management of pain. By improving venous localization and reducing the number of needle passes, these techniques minimize procedural trauma and the cumulative pain experience, which is a crucial, though indirect, component of non-pharmacological analgesia. Furthermore, distraction-based interventions, including aromatherapy, guided breathing, and music therapy, effectively attenuate procedural anxiety and stabilize hemodynamic parameters. This appears to correlate with enhanced patient-reported satisfaction with nursing care. Given their high safety profile and cost-effectiveness, these non-pharmacological methods could be considered for broader implementation in clinical practice to support the management of procedural pain, although further high-quality research is needed to confirm the strength of these conclusions.

## 7. Implications for Clinical Practice

The findings of this systematic review suggest that non-pharmacological modalities offer a highly feasible and effective strategy for procedural pain management, which should be integrated into standard nursing protocols. Given their rapid onset of action and excellent safety profiles, mechanical and thermal interventions (e.g., vibration-cold devices and active warming) are particularly suited for clinical environments requiring high time-efficiency. Furthermore, the implementation of low-cost behavioral techniques, such as guided breathing and distraction, provides a universal baseline for analgesia that is accessible regardless of institutional resources.

In the context of modern clinical practice, these results advocate for the broader inclusion of non-pharmacological pain-reduction tools in nursing curricula and hospital guidelines. Such an approach empowers nursing staff to independently enhance patient comfort, bypassing the regulatory constraints and administrative requirements often associated with the delivery of pharmacological agents.

The consistent finding of in the majority of the analyzed RCTs, paired with large effect sizes for VR and thermomechanical devices, suggests that these interventions provide the most reliable and clinically significant analgesic outcomes for adult patients undergoing peripheral venous cannulation.

Based on the synthesis of current evidence, we propose a stepped-care “Clinical Pathway” for non-pharmacological pain management during peripheral intravenous cannulation (PIVC). This approach prioritizes interventions based on their ease of implementation, minimal resource requirements, and patient acceptance (Table 8).

## Figures and Tables

**Table 1:** Search strategies.

Databases	Search Strategy
PubMed	(“Catheterization, Peripheral”[Mesh] OR “Venipuncture”[Mesh] OR “peripheral venous cannulation” OR “intravenous cannulation” OR “IV cannulation” OR “peripheral catheter *” OR “short peripheral catheter *” OR “PIVC” OR “IV insertion”) AND (“Pain”[Mesh] OR “Anxiety”[Mesh] OR “pain” OR “anxiety” OR “discomfort” OR “analgesi *”) AND (“Cryotherapy”[Mesh] OR “Aromatherapy”[Mesh] OR “non-pharmacological” OR “distraction” OR “virtual reality” OR “VR” OR “music” OR “vibration” OR “buzzy” OR “aromatherapy” OR “essential oil *” OR “breathing” OR “cough” OR “massage” OR “cutaneous stimulation” OR “audiovisual” OR “hypnosis” OR “heat” OR “warming” OR “thermal” OR “cold pack” OR “ice pack” OR “cryotherapy” OR “stress ball” OR “spirometer” OR “vein visualization” OR “near-infrared” OR “NIR” OR “ultrasound”) NOT (“Child”[Mesh] OR “Infant”[Mesh] OR “child *” OR “pediatric *” OR “paediatric *” OR “infant *” OR “neonate *” OR “newborn” OR “lidocaine” OR “EMLA” OR “tetracaine” OR “picc” OR “midline” OR “central venous” OR “dialysis” OR “fistula” OR “arterial” OR “spray” OR “vapocoolant” OR “aerosol”) Limit: years, adults, language Results: 164
CINAHL	((MH “Catheterization, Peripheral+”) OR (MH “Venipuncture+”) OR TX “peripheral venous cannulation” OR TX “intravenous cannulation” OR TX “IV cannulation” OR TX “peripheral catheter *” OR TX “short peripheral catheter *” OR TX “PIVC” OR TX “IV insertion”) AND ((MH “Pain+”) OR (MH “Anxiety+”) OR TX “pain” OR TX “anxiety” OR TX “discomfort” OR TX “analgesi *”) AND (TX “non-pharmacological” OR TX “distraction” OR TX “virtual reality” OR TX “VR” OR TX “music” OR TX “vibration” OR TX “buzzy” OR (MH “Aromatherapy+”) OR TX “aromatherapy” OR TX “essential oil *” OR TX “breathing” OR TX “cough” OR TX “massage” OR TX “cutaneous stimulation” OR TX “audiovisual” OR TX “hypnosis” OR TX “heat” OR TX “warming” OR TX “thermal” OR TX “cold pack” OR TX “ice pack” OR (MH “Cryotherapy+”) OR TX “cryotherapy” OR TX “stress ball” OR TX “spirometer” OR TX “vein visualization” OR TX “near-infrared” OR TX “NIR” OR TX “ultrasound”) NOT ((MH “Child+”) OR (MH “Infant+”) OR TI (child * OR pediatric * OR paediatric * OR infant * OR neonate * OR newborn) OR TX lidocaine OR TX EMLA OR TX tetracaine OR TX picc OR TX midline OR TX “central venous” OR TX “dialysis” OR TX “fistula” OR TX “arterial” OR TX spray OR TX vapocoolant OR TX aerosol) Limit: years, adults Results: 13
Web of Science	TS = ((“peripheral venous cannulation” OR “intravenous cannulation” OR “IV cannulation” OR “peripheral catheter *” OR “short peripheral catheter *” OR “venipuncture” OR “venous access” OR “PIVC” OR “IV insertion”)AND (“pain” OR “anxiety” OR “discomfort” OR “analgesi *”)AND(“non-pharmacological” OR “distraction” OR “virtual reality” OR “VR” OR “music” OR “vibration” OR “buzzy” OR “aromatherapy” OR “essential oil *” OR “breathing” OR “cough” OR “massage” OR “cutaneous stimulation” OR “audiovisual” OR “hypnosis” OR “heat” OR “warming” OR “thermal” OR “cold pack” OR “ice pack” OR “cryotherapy” OR “stress ball” OR “spirometer” OR “vein visualization” OR “near-infrared” OR “NIR” OR “ultrasound”))NOT TI = (child * OR pediatric * OR paediatric * OR infant * OR neonate * OR newborn)NOT TS = (lidocaine OR EMLA OR tetracaine OR picc OR midline OR “central venous” OR dialysis OR fistula OR arterial OR spray OR vapocoolant OR aerosol) Limit: years Results: 96
Scopus	TITLE-ABS-KEY (“peripheral venous cannulation” OR “intravenous cannulation” OR “IV cannulation” OR “peripheral catheter *” OR “short peripheral catheter *” OR “venipuncture” OR “venous access” OR “PIVC” OR “IV insertion”) AND (“pain” OR “anxiety” OR “discomfort” OR “analgesi *”) AND(“non-pharmacological” OR “distraction” OR “virtual reality” OR “VR” OR “music” OR “vibration” OR “buzzy” OR “aromatherapy” OR “essential oil *” OR “breathing” OR “cough” OR “massage” OR “cutaneous stimulation” OR “audiovisual” OR “hypnosis” OR “heat” OR “warming” OR “thermal” OR “cold pack” OR “ice pack” OR “cryotherapy” OR “stress ball” OR “spirometer” OR “vein visualization” OR “near-infrared” OR “NIR” OR “ultrasound”)) AND NOT TITLE-ABS-KEY (“child *” OR “pediatric *” OR “paediatric *” OR “infan t*” OR “neonate *” OR “newborn” OR “lidocaine” OR “EMLA” OR “tetracaine” OR “picc” OR “midline” OR “central venous” OR “dialysis” OR “fistula” OR “arterial” OR “spray” OR “vapocoolant” OR “aerosol”) Limit: years, language Results: 88

PIVC—Peripheral Intravenous Catheter, IV—intravenously.

**Table 2:** PICO framework.

	Inclusion Criteria
Population (P)	Adult patients ≥ 18 years old
Intervention (I)	To relief pain during cannulation
Comparison (C)	Standard/usual care
Outcome (O)	Procedural pain intensity (primary outcome), anxiety levels, patient satisfaction, first-attempt success rate, safety/adverse events.

**Table 3:** Inclusion and exclusion criteria.

	Inclusion Criteria	Exclusion Criteria
Patients	Adult patients (≥18 years old), Inpatient, outpatient	Pediatric patients (<18 years old),
Intervention	Non-pharmacological (e.g., warming, cooling, music),	Pharmacological interventions (vapocoolant spray (ethyl chloride))
Type of Catheter	PIVC only, (SPC)	Midline, PICC, CVC, HDCVC, Port
Years considered/Time period	All evidence published in the last 10 years, period 2015–2025	Publications prior to 2015
Language	English, Polish	Other languages
Databases	PubMed, CINAHL, Web of Science, Scopus	Other databases, Gray literature,
Study Type	RCTs, Quasi-experimental Prospective/Retrospective	Quantitative studies Qualitative studies Reviews (any types) Letters to the editor Case reports

PIVC—Peripheral Intravenous Catheter, SPC-Short Peripheral Catheter, PICC-Peripherally Inserted Central Catheter, CVC-Central Venous Catheter, HDCVC-Hemodialysis Central Venous Catheter.

**Table 4:** JBI Critical Appraisal Tools—checklist for randomized controlled trials.

Author, Year	1	2	3	4	5	6	7	8	9	10	11	12	13	Total	
Junges et al. (2024) [12]	Y	Y	Y	N	N	Y	Y	Y	Y	Y	Y	Y	Y	11/13	H
Çetin et al. (2019) [13]	Y	U	Y	N	N	N	Y	Y	U	Y	Y	Y	Y	8/13	L
Basak et al. (2020) [14]	Y	U	Y	N	N	U	Y	Y	N	Y	Y	Y	Y	8/13	L
Özkan et al. (2024) [15]	Y	U	Y	N	N	N	Y	Y	N	Y	Y	Y	Y	8/13	L
Redfern et al. (2018) [16]	Y	U	Y	N	N	U	Y	Y	Y	Y	Y	Y	Y	9/13	M
Yılmaz et al. (2018) [17]	Y	U	Y	N	N	U	Y	Y	N	Y	Y	Y	Y	8/13	L
Yılmaz et al. (2024) [18]	Y	U	Y	N	N	Y	Y	Y	Y	Y	Y	Y	Y	10/13	M
Rişvan et al. (2025) [19]	Y	U	Y	N	N	U	Y	Y	N	Y	Y	Y	Y	8/13	L
Özbay et al. (2025) [20]	Y	U	Y	N	N	U	Y	Y	N	Y	Y	Y	Y	8/13	M
Simón-López et al. (2020) [21]	Y	Y	Y	N	N	Y	Y	Y	N	Y	Y	Y	Y	10/13	M
Serin et al. (2025) [22]	Y	U	Y	N	N	N	Y	Y	Y	Y	Y	Y	Y	9/13	M
Yılmaz et al. (2025) [23]	Y	U	Y	N	N	U	Y	Y	Y	Y	Y	Y	Y	9/13	M
Fusco et al. (2020) [24]	Y	Y	Y	N	N	Y	Y	Y	Y	Y	Y	Y	Y	11/13	H
Erzincanli et al. (2021) [25]	N	N	Y	N	N	U	Y	Y	N	Y	Y	Y	Y	7/13	L
Kaplan et al. (2024) [26]	Y	U	Y	N	N	U	Y	Y	N	Y	Y	Y	Y	8/13	M
Hosseini et al. (2025) [27]	Y	Y	Y	N	N	U	Y	Y	N	Y	Y	Y	Y	9/13	M
Corbasson et al. (2025) [28]	Y	Y	Y	N	N	N	Y	Y	Y	Y	Y	Y	Y	10/13	M
Lee, Her, & Hur (2025) [29]	Y	U	Y	N	N	Y	Y	Y	N	Y	Y	Y	Y	9/13	M
Karaman et al. (2016) [30]	Y	U	Y	U	N	Y	Y	Y	Y	Y	Y	Y	Y	10/13	M
Sharma et al. (2024) [31]	N	N	N	N	N	N	Y	Y	Y	Y	Y	Y	Y	7/13	L
Akçoban & Tosun (2025) [32]	Y	U	Y	N	N	Y	Y	Y	Y	Y	Y	Y	Y	10/13	M
Hur & Choi (2021) [33]	Y	U	Y	N	N	U	Y	Y	Y	Y	Y	Y	Y	9/13	M
Berna et al. (2024) [34]	Y	Y	Y	Y	U	Y	Y	Y	N	Y	Y	Y	Y	11/13	H
Hosseinabadi et al. (2015) [35]	Y	U	Y	Y	N	U	Y	Y	Y	Y	Y	Y	Y	10/13	M
Basak et al. (2024) [36]	Y	U	Y	N	N	Y	Y	Y	N	Y	Y	Y	Y	9/13	M
Jisha et al. (2017) [37]	Y	U	Y	N	N	U	Y	Y	Y	Y	Y	Y	Y	9/13	M
Fumagalli et al. (2017) [38]	Y	U	Y	N	N	N	Y	Y	Y	Y	Y	Y	Y	9/13	M
Tapar et al. (2018) [39]	Y	Y	Y	N	N	Y	Y	Y	N	Y	Y	Y	Y	10/13	M
Guillon et al. (2015) [40]	N	N	Y	N	N	N	Y	Y	Y	Y	Y	Y	N	7/13	L
Rodríguez-Herrera et al. (2022) [41]	N	N	Y	N	N	N	Y	Y	Y	Y	Y	Y	Y	8/13	L

Y—Yes, N—No, U—unclear, U—unclear; H = High (if ≥80% of the assessment tool items received a point), M = Moderate (if ≥65% of the assessment tool items received a point); L = Low ≤ 55% (if ≤55% of the assessment tool items received a point).

**Table 5:** JBI Critical Appraisal Tools—checklist for quasi-experimental studies.

Author, Year	1	2	3	4	5	6	7	8	9	Total
Usichenko et al. (2019) [42]	Y	Y	Y	Y	Y	Y	Y	Y	Y	9/9

Y—Yes, N—No, U—unclear, N/A—not applicable, U—unclear; H = High (if ≥80% of the assessment tool items received a point); M = Moderate (if ≥65% of the assessment tool items received a point); L = Low ≤ 55% (if ≤55% of the assessment tool items received a point.

**Table 6:** Charakteristic of included studies and results.

Author (Year)	Study Design	Sample Sizes	Group Sizes	Setting	Intervention	Pain Assessment Tool	Pain Level	Results
Junges et al. (2024) [12]	A randomized clinical trial	N = 64	IG n = 32 CG n = 32	Public hospital in southern Brazil	Intervention group (IG/Experimental): US-guided peripheral venipuncture performed by specialist nurses of a Vascular Access Team (VAT) Control group (CG): Conventional peripheral venipuncture (palpation/visualization) performed by clinical practice nurses	NRS	IG (US-guided, specialist nurses): Pain NRS = 2 CG (Conventional): Pain NRS = 4	+ Patients undergoing US-guided peripheral venipuncture experienced less procedural pain than those in the conventional group (median NRS 2 vs. 4, *p* < 0.001).
Çetin et al. (2019) [13]	A randomized controlled, pretest and post-test experimental study.	N = 100	IG n = 50 CG n = 50	University hospital in Turkey	Application of the Buzzy^®^ device (combined vibration and cold gel pack) during peripheral intravenous catheterization to reduce pain and anxiety.	VAS	Mean VAS score was 1.04 ± 0.96 cm in the Buzzy (intervention) group and 5.32 ± 1.64 cm in the control group (*p* < 0.001).	+ Use of the Buzzy device was associated with a significant reduction in pain intensity during peripheral intravenous catheterization compared with standard care (*p* < 0.001).
Basak et al. (2020) [14]	A randomized controlled trial	N = 120	IG1 n = 30: VR (3D video) IG2 n = 30: Optical illusion pictures CG n = 30: Standard procedure	Gülhane Training and Research Hospital Turkey	Use of distractive methods (cards with optical illusions and 3D videos via VR goggles) during peripheral intravenous catheter insertion to reduce pain and increase patient satisfaction.	VAS	4.72 ± 3.15 (control) vs. 3.32–3.50 ± 2.81–2.84 (distraction groups)	+ Distraction methods (cards and 3D VR videos) during PIC insertion significantly reduced pain (VAS 3.32–3.50 vs. 4.72; *p* = 0.02) and increased patient satisfaction (8.07 vs. 5.12; *p* = 0.01) compared with control.
Özkan et al. (2024) [15]	A randomized controlled trial	N = 130	IG n = 65 CG n = 65	General Surgery Clinic of a university hospital in Turkey.	Diaphragmatic breathing exercises performed during peripheral venous catheterization	NRS	Intervention group: 0.29 ± 0.70; Control group: 1.30 ± 1.47	+ Intervention significantly reduced pain and anxiety, increased patient satisfaction (Pain: 0.29 vs. 1.30; Anxiety: 3.18 vs. 4.35; Satisfaction: 7.66 vs. 4.87), and decreased pulse rate, blood pressure, and procedure time
Redfern et al. (2018) [16]	A Prospective Randomized Study	N = 105	IG n = 49 CG n = 56	hospital in Toledo, Ohio, USA	The experimental group received thermomechanical stimulation using the Buzzy device (combined cold and vibration applied approximately 5 cm above the IV insertion site), while the control group received standard IV catheter insertion without any pain-reducing intervention.	VAS	IG: Mean 2.52 CG: Mean 2.43 (No significant difference, *p* = 0.86)	− Thermomechanical stimulation did not significantly reduce overall procedural pain or improve patient satisfaction compared with standard care; however, patients with higher preprocedural anxiety experienced lower pain scores when the Buzzy device was used, suggesting a potential benefit in highly anxious individuals.
Yılmaz et al. (2018) [17]	A single blind randomized controlled study.	N = 120	IG1 n = 30 IG2 n = 30 IG3 n = 30 CG n = 30	The blood center of a university hospital in the Marmara region, Turkey.	Interventions included coughing, blowing into a spirometer, squeezing a stress ball, and no additional intervention in the control group.	VAS	Mean pain scores were: Coughing: 19.5 mm, Spirometer: 28.3 mm, Stress ball: 32.1 mm, Control: 45.5 mm.	+ All three interventions significantly reduced pain compared to the control group (*p* < 0.001), with the greatest effect observed in the coughing group; no statistically significant differences were found between the experimental groups.
Yılmaz et al. (2024) [18]	A randomized controlled trial	N = 120	IG1 n = 30: Local heat IG2 n = 30: Local cold IG3 n = 30: Vibration (Buzzy) CG n = 30: Standard procedure	Yellow and green area of the emergency unit of a university hospital in the Marmara region of Turkey	Heat, cold, or vibration applied before peripheral intravenous catheter insertion to reduce pain.	VAS	Cold group: 30.50 ± 16.78; Heat group: 48.66 ± 17.16; Vibration group: 56.66 ± 14.03; Control: 48.36 ± 24.02	+/− Only cold application significantly reduced pain (*p* = 0.002) and shortened procedure duration (*p* = 0.001). Heat and vibration did not significantly reduce pain compared to the control group (*p* > 0.05).
Rişvan et al. (2025) [19]	A randomized controlled trial	N = 111	IG1 n = 37: VR glasses IG2 n = 37: Ball squeezing CG n = 37: Standard procedure	University hospital in Turkey	VR simulation or ball-squeezing during peripheral cannula placement	NRS	VR group: 1.94 ± 0.40 Ball squeezing group: 0.85 ± 0.23 Control group: 2.80 ± 0.36	+ Both interventions reduced pain compared with control; VR NRS = 1.94, Ball-squeezing NRS = 0.85, Control NRS = 2.80
Özbay et al. (2025) [20]	A randomized controlled study	N = 124	IG1 n = 31: Cough trick IG2 n = 31: Spirometer blowing IG3 n = 31: Stress ball CG n = 31: Standard procedure	The study was conducted in the emergency department (yellow zone) of a hospital in Turkey	The interventions included the cough trick, spirometer blowing, and stress ball squeezing applied during peripheral intravenous catheterization, while the control group received standard PIVC without any nonpharmacological intervention.	NRS	IG1: Mean 1.48 ± 1.28 IG2: Mean 2.29 ± 1.63 IG3: Mean 3.16 ± 1.77 CG: Mean 4.48 ± 2.14	+ All three nonpharmacological interventions significantly reduced pain and anxiety levels and improved comfort during peripheral intravenous catheterization compared with the control group (*p* < 0.05).
Simón-López et al. (2020) [21]	A randomized controlled trial	N = 59	Dry topical heat: n = 21 High pressure: n = 18 Combined heat and pressure: n = 20.	Clinical Trial Unit of Hospital Universitario de La Princesa in Madrid, Spain.	The interventions consisted of dry topical heat applied for 7 min, controlled high pressure at 100 mmHg using a sphygmomanometer cuff, and a combination of heat and pressure, compared with standard tourniquet application as the control.	VAS	The authors emphasize that a clinically significant reduction in pain was defined as a decrease of at least 1 point on the VAS, and this effect was achieved with the high-pressure intervention and the combined heat and pressure intervention, even though the absolute pain level remained relatively high.	+ All three interventions were significantly more effective than standard practice in achieving first-attempt peripheral venous cannulation, with high pressure being the most effective and least painful, and with no increase in haemolysis.
Serin et al. (2025) [22]	A randomized controlled trial	N = 126	Thermomechanical stimulation group n = 42, Virtual reality glasses group n = 42, Control group n = 42	Emergency department of Etimesgut Şehit Sait Ertürk State Hospital, Ankara, Turkey.	Cold vibrating thermomechanical device, Virtual reality glasses showing nature video, Control (no intervention)	VAS	VR: 2.71 ± 1.45, Thermomechanical: 2.66 ± 1.22, Control: 4.85 ± 1.71	+ Both thermomechanical stimulation and VR glasses significantly reduced pain and increased satisfaction compared to control (*p* < 0.05); no significant difference between the two experimental groups (*p* > 0.05)
Yılmaz et al. (2025) [23]	A randomized controlled trial	N = 152	NIR light group n = 50; transilluminator group n = 50; control group n = 52	Oncology outpatient chemotherapy unit, tertiary hospital, Turkey	Peripheral IV cannulation using near-infrared (NIR) vein visualization or transilluminator versus standard method.	VAS	NIR group: Median 2.0 (IQR: 1.0–3.0). Transilluminator group: Median 3.0 (IQR: 2.0–4.0). Control group: Median 2.0 (IQR: 1.0–4.0)	− No significant effect of NIR or transilluminator on pain or fear; first-attempt success highest in control (96.2%) and NIR (90%); satisfaction higher in NIR and control than transilluminator; procedure time longest with transilluminator
Fusco et al. (2020) [24]	A multicenter randomized trial	N = 272	Hypnosis group: n = 89 Nocebo group: n = 91 Neutral group: n = 92	Multicenter study in hospitals in France and Belgium	The study compared three types of structured communication during peripheral intravenous cannulation: a hypnosis group using a confusion technique and positive suggestions, a neutral group using factual descriptions of the procedure, and a nocebo group using words with negative connotations like “sting” or “pain”	NRS	Hypnosis group: Mean NRS = 1.5 ± 1.9 (range 0–5) Neutral group: Mean NRS = 3.5 ± 2.3 (range 0–9) Nocebo group: Mean NRS = 3.8 ± 2.5 (range 0–10)	+ Pain after PIVC was significantly lower in the hypnosis group compared with neutral and nocebo groups (hypnosis mean NRS 1.5 vs neutral 3.5 and nocebo 3.8, *p* < 0.0001).
Erzincanli et al. (2021) [25]	A Randomized Controlled Trial	N = 97	Hand massage group n = 48 CG n = 49	Blood collection room of a training and research hospital in the inner region of Turkey	5–6-min hand massage applied to both hands and arms before venipuncture	VAS	No significant difference (*p* > 0.05); Post-procedure VAS median: Experimental 0 [0–8] vs. Control 1 [0–3]	− Hand massage significantly reduced anxiety (*p* < 0.05) and lowered systolic/diastolic blood pressure and heart rate (*p* < 0.05), but did not significantly affect pain levels (*p* > 0.05)
Kaplan et al. (2024) [26]	A prospective randomized study	N = 200	Participants were divided into four groups: Forearm 20 Ga n = 50, Forearm 22 Ga n = 50, Dorsum of the hand 20 Ga n = 50, and Dorsum of the hand 22 Ga n = 50.	The emergency department of a university hospital in Turkey.	Peripheral intravenous catheterization was performed using 20 Ga or 22 Ga catheters in either the forearm or the dorsum of the hand.	VAS	The mean VAS score was lowest for 22 Ga in the forearm (8.92 ± 9.81 mm) and highest for 20 Ga in the dorsum of the hand (39.48 ± 8.83 mm).	+ Smaller catheters (22 Ga) inserted in the forearm resulted in significantly lower pain levels and higher patient satisfaction compared to other groups.
Hosseini et al. (2025) [27]	A randomized clinical trial	N = 99	VM group: n = 33; Auriculotherapy group: n = 33; Control group: n = 33	Emergency department of Allama Bahloul Gonabadi Hospital in Gonabad, Iran	VM group: Exhaling against a closed glottis for 20 s while pressing the nose tip. Auriculotherapy group: Pressure applied to a specific earlobe point using a probe for 2 min. Control group: Standard PIVC procedure with disinfection and 20-gauge catheter.	VAS	VM group: 2.88 ± 3.81. Auriculotherapy group: 2.57 ± 3.45. Control group: 7.13 ± 2.39	+ There was a significant difference between intervention groups and control (*p* < 0.001). No significant difference was found between VM and auriculotherapy (*p* = 0.67)
Corbasson et al. (2025) [28]	A randomized clinical trial	N = 126	IG n = 65 CG n = 61	Two tertiary care reference centers in France (HEGP and Henri Mondor Hospital)	Use of the AccuVein AV400 (near-infrared visualization) for peripheral IV placement	VAS	Significantly lower in the AccuVein group: Median VAS: 2.5 (n = 65) vs. Median VAS: 4 (n = 61); *p* = 0.02.	+ No significant difference in attempts (Median: 2 for both groups; *p* = 0.49). However, the device notably reduced procedural pain for the patients.
Lee, Her, & Hur (2025) [29]	Randomized controlled trial	N = 118	Heat therapy group: n = 29 Cold therapy group: n = 29 TGI group (Thermal Grill Illusion—alternating thermal stimuli): n = 30 CG: n = 30	Daejeon Eulji Medical Center, South Korea	Participants received venipuncture with a TEE tourniquet applied near the puncture site, using either heat therapy (40–45 °C), cold therapy (0–10 °C), alternating heat/cold for TGI therapy, or no thermal activation for the control group.	NRS	Perceived pain levels during venipuncture were 3.31 ± 1.65 for heat therapy, 4.24 ± 1.33 for cold therapy, 4.10 ± 1.92 for TGI therapy, and 5.00 ± 1.88 for the control group.	Heat therapy significantly reduced perceived pain (*p* = 0.003) and stress (*p* = 0.004) compared to control; no significant differences were found for SpO2 or satisfaction
Karaman et al. (2016) [30]	Prospective, randomized, placebo-controlled study.	N = 101	Lavender group n = 51 CG n = 50	Preoperative holding area of a university hospital, Turkey.	The lavender group inhaled two drops of 1% lavender essential oil from a gauze pad for 5 min before and during cannulation, while the control group inhaled pure water	VAS	The mean pain score was significantly lower in the lavender group at 1.94 compared to 2.48 in the control group (*p* = 0.01)	+ Lavender aromatherapy significantly reduced pain (*p* = 0.01) and anxiety (*p* < 0.001) while significantly improving patient satisfaction (*p* = 0.003) compared to the placebo
Sharma et al. (2024) [31]	Experimental research with a pretest-posttest design	N = 60	IG n = 30 CG n = 30	General wards of Mahatma Gandhi Hospital, Jaipur, Rajasthan, India.	Local moist heat therapy applied for 10 min using a folded towel soaked in 40∘C water at the selected vein site before cannulation	NRS	The interventional group reported significantly lower pain (1.56 ± 1.79) compared to the standard group (5.13 ± 2.47)	+ Local moist heat application significantly improved vein visibility and palpability (4.1 ± 0.7 vs. 2.36 ± 0.6), reduced patient pain perception (1.56 ± 1.79 vs. 5.13 ± 2.47), and increased first-attempt success rates to 96.66% while significantly decreasing the time required for cannulation
Akçoban & Tosun (2025) [32]	A randomized, controlled, single-blind clinical trial.	N = 138	Valsalva group: n = 46 Breathing exercise group: n = 46 CG n = 46	The general surgery unit of a state hospital in Turkey	1. Valsalva Group: Patients blew into a tube connected to a manometer, maintaining 30 mmHg for 20 s before catheterization 2. Breathing Exercise Group: Rhythmic breathing (3 s inhale, 3 s hold, 3 s exhale) starting two minutes before the procedure. 3. Control Group: Received routine peripheral intravenous catheterization (PIVC) without additional intervention.	VAS	During the procedure, mean VAS scores were significantly lower in the Valsalva (28.40 ± 17.80) and Breathing (27.20 ± 16.90) groups compared to the Control (48.20 ± 18.90) group (*p* < 0.001).	+ Both interventions significantly reduced pain compared to the control group (*p* < 0.001), with no statistically significant difference found between the two active intervention groups (*p* > 0.05). Additionally, both methods helped regulate pulse rates post-procedure.
Hur & Choi (2021) [33]	A randomized controlled trial	N = 120	Heat therapy n = 30, Cold therapy n = 30, Thermal Grill Illusion (TGI) therapy n = 30, CG n = 30	Healthcare center in South Korea	Application of a flexible Thermoelectric Element (TEE) band for 10 s during venipuncture. Interventions included: heat (40–45∘C), cold (0–10∘C), or TGI (simultaneous 40–45∘C and 0–10 ∘C). The control group wore the band without stimulation	NRS	Post-intervention means: Heat (3.30), Cold (2.71), TGI (3.96), Control (3.57)	+ No significant differences were found between groups for subjective pain (*p* = 0.173), anxiety (*p* = 0.327), or physiological responses. However, there was a significant difference in satisfaction (F = 4.21, *p* = 0.007), with the cold therapy group reporting the highest satisfaction
Berna et al. (2024) [34]	A randomized controlled trial	N = 251	Phase 1 (Audio): Control n = 56, Intervention n = 57. Phase 2 (Nurse): Control n = 54, Intervention n = 58.	Emergency Department (ED) of a tertiary teaching hospital in Lausanne, Switzerland.	Positive vs. Standard verbal suggestions regarding peripheral intravenous catheter (PIC) placement. Phase 1: Messages delivered via Bluetooth headset (audio recording). Phase 2: Messages delivered by trained nurses performing the procedure.	VAS	Patients reported relatively low procedural pain (median VAS scores ranging from 9 to 22 mm). This occurred in a context where patients already had moderate pain due to their arrival condition.	− The intervention did not significantly affect pain or anxiety reports in either phase (all *p* > 0.2). While nurses showed higher empathic behavior in the positive message group of Phase 2 (*p* < 0.001), this did not translate to clinical relief for the patients
Hosseinabadi et al. (2015) [35]	Double blind clinical trial	N = 187	Acupressure group n = 60, PG n = 66, CG n = 61	Hospital in Khorramabad, Iran	Massage of specific acupoints (LI 4, Extra 1, and Shenmen) for 6 min (two 3-min cycles) prior to and during venipuncture. Placebo group received massage on false points, and control group received routine care.	VAS	Post-intervention mean pain scores: Acupressure (2.42), Placebo (3.27), Control (3.26)	+ Acupressure significantly reduced venipuncture pain compared to placebo (*p* = 0.01) and control (*p* = 0.004) groups. Anxiety scores decreased significantly within the acupressure (*p* = 0.002) and placebo (*p* = 0.029) groups after intervention, but no significant differences were found between the three groups regarding anxiety or vital signs post-intervention (*p* > 0.05).
Basak et al. (2024) [36]	Convergent parallel mixed-method design	N = 126	Rose oil group n = 42, Hand-holding group n = 42, CG n = 42	Endoscopy and colonoscopy units of a state hospital in Ankara, Turkey	Rose oil group: Inhalation of two drops of rose oil (Rosa Damascena Mill.) applied to a surgical mask for 2 min before PIVC insertion. Hand-holding group: A family member held the patient’s hand from the start until the end of the procedure. Control group: Standard PIVC insertion procedure with no pain-reduction intervention	NRS	Rose oil group: 2.40 ± 1.78 (Mild) Hand-holding group: 3.53 ± 1.98 (Moderate) Control group: 4.88 ± 1.56 (Moderate)	+ There was a statistically significant difference in pain scores between the groups (*p* = 0.001). Both interventions reduced pain compared to the control group but rose oil aromatherapy was significantly more effective than handholding. Qualitative data revealed themes of “Pain and fear experiences” and “Satisfaction with the interventions”.
Jisha et al. (2017) [37]	True experimental post-test only with control group design	N = 60	Dry Heat (Group I n = 20), Moist Heat (Group II n = 20), and Control Group n = 20	A selected hospital at Mangalore, India	Dry Heat: Hot water bag (120–140 °F) for 7 min. Moist Heat: Moist towel (110–115 °F) for 7 min.	NRS	Results showed Median = 1 for Dry Heat, Median = 5 for Moist Heat, and Median = 7 for Control.	+ Dry heat is more effective than moist heat, resulting in the least time for cannulation (Median = 1) and the least pain (Median = 1).
Fumagalli et al. (2017) [38]	Pilot randomized study	N = 103	Standard technique: n = 56; NIR-BD: n = 47	Intensive Care Unit (ICU), University of Florence, Italy	Venous puncture guided by a Near-Infrared (NIR) device (Easy Vein)	VAS	No significant difference; VAS score ≥ 1 in 44.6% (Standard) vs. 51.1% (NIR-BD)	NIR-BD significantly reduced hematomas and lowered anxiety/depression scores without increasing procedure time
Tapar et al. (2018) [39]	Prospective, randomized controlled study	N = 150	Group M n = 50, Group V n = 50, and Group C n = 50	Preoperative care room at Gaziosmanpasa University School of Medicine Hospital, Tokat, Turkey	Group M: Listened to self-selected music via MP3 player for 5 min during the procedure. Group V: Performed the Valsalva maneuver (deep inhale and breath-hold for up to 20 s) during cannulation. Group C: No intervention during the procedure.	VAS	Group M: 3.20 ± 0.92 Group V: 3.41 ± 0.74 Group C: 3.94 ± 1.30	+ Both music and the Valsalva maneuver significantly reduced pain compared to the control (*p* < 0.05). However, only music significantly reduced anxiety (*p* = 0.003) and increased patient satisfaction (*p* = 0.004) compared to the control group.
Guillon et al. (2015) [40]	Prospective multicenter evaluation with 1:1 alternating assignment	N = 450	Total: 229 used the NIR visualizer; 221 did not DVA Status: 165 patients with Difficult Venous Access (DVA); 285 non-DVA patients	Four French Hemophilia Treatment Centers (HTCs) located in Caen, Paris, Lille, and Reims	Use of a near-infrared (NIR) vein visualizer (AccuVein Vein Viewing System) to map superficial veins in real-time on the skin	NRS	Measured on a scale of 0 to 10 (0 = no pain, 10 = severe pain). Pain was significantly less common in DVA patients using the device (34.0% vs. 55.4%; *p* = 0.019)	+ DVA affected 36.7% of patients. In DVA patients, the NIR device significantly reduced difficulty in locating veins (76% vs. 92.3%) and reduced pain, though it did not affect the number of puncture attempts
Rodríguez-Herrera et al. (2022) [41]	Case–control study, randomized research	N = 72	Cases (UST): 34; Control (ST): 38	Emergency services unit of a level III hospital (Spain)	Ultrasound-guided technique (UST) vs. Standard technique (ST)	VAS	Mean VAS: 4.58 (UST) vs. 6.55 (ST)	UST decreased punctures (1.23 vs. 2.92), reduced time (126 s vs. 618 s), and lowered pain (4.58 vs. 6.55)
Usichenko et al. (2019) [42]	Prospective experimental study	N = 54	Investigation 1: n = 21 Investigation 2: n = 20 Investigation 3: n = 13	Laboratory of the University Medical Center Greifswald	Investigation 1 (IG): Cough-trick (CT)—participants coughed during venipuncture Investigation 1 (CG): Weak distraction—squeezing a rubber ball during venipuncture Investigation 2 (IG): Cough-trick (CT)—participants coughed during venipuncture Investigation 2 (CG): Strong distraction—inflating tourniquet to 200 mmHg during venipuncture Investigation 3 (IG): Cough-trick (CT) under saline—participants coughed during venipuncture Investigation 3 (CG): Cough-trick (CT) under naloxone/no intervention—venipuncture without pain-modifying effect	VAS	Inv 1: CT 27 mm vs. Weak 32 mm; Inv 2: CT 28 mm vs. Strong 25 mm; Inv 3: CT + Naloxone 26 mm vs. No intervention 23 mm	+ CT is superior to weak distraction (*p* = 0.03) and equivalent to strong distraction (*p* = 0.3); its analgesic effect is abolished by naloxone

Legend: NRS: Numerical Rating Scale; VAS: Visual Analog Scale; VRS: Verbal Rating Scale; [+]: Positive effect (significant pain reduction); [−]: No effect (no statistically significant difference), IG: Intervention group; CG: Control group.

**Table 7:** Summary of evidence for non-pharmacological interventions during PIVC.

Intervention Category	Evidence Strength	Quality (JBI Score)	Clinical Consistency
Thermomechanical (Buzzy^®^)	Strong	High	High
Virtual Reality (VR)	Moderate/Strong	High	Moderate
Active Warming	Moderate	Moderate	Moderate
Behavioral (Cough/Breathing)	Moderate	Moderate	Moderate
Aromatherapy	Low/Weak	Low	Low
Music Therapy	Low/Weak	Low	Moderate

**Table 8:** Clinical Pathway for Non-Pharmacological Pain Management during PIVC.

Step	Intervention Category	Specific Techniques	Best For	Implementation Priority
1	Behavioral	Cough trick [14,17], Diaphragmatic breathing [12]	All patients, especially those with high anxiety	High (First line)
2	Distraction	Music therapy, Smartphone-based distraction (video/games)	Patients with moderate procedural anxiety	High
3	Physical	Vibration/Cold therapy (e.g., Buzzy^®^) [10,15,19]	Patients with low pain threshold	Moderate
4	Advanced	Virtual Reality (VR) [11,16,19], NIR vein visualization	Patients with anticipated difficult access or chronic needle phobia	Low (Resource-dependent)

## Data Availability

The authors declare that the data of this research are available from the correspondence author on request.

## Supplementary Materials

The following supporting information can be downloaded at: https://www.mdpi.com/article/10.3390/jcm15072662/s1, PRISMA Checklist. Reference [11] is cited in the Supplementary Materials.
